# Epidemiology and morbidity of hookworm-related cutaneous larva migrans (HrCLM): Results of a cohort study over a period of six months in a resource-poor community in Manaus, Brazil

**DOI:** 10.1371/journal.pntd.0006662

**Published:** 2018-07-19

**Authors:** Felix Reichert, Daniel Pilger, Angela Schuster, Hannah Lesshafft, Silas Guedes de Oliveira, Ralf Ignatius, Hermann Feldmeier

**Affiliations:** 1 Charité–Universitätsmedizin Berlin, corporate member of Freie Universität Berlin, Humboldt-Universität zu Berlin, and Berlin Institute of Health, Institute of Microbiology and Infection Immunology, Berlin, Germany; 2 Charité–Universitätsmedizin Berlin, corporate member of Freie Universität Berlin, Humboldt-Universität zu Berlin, and Berlin Institute of Health, Department of Neonatology, Berlin, Germany; 3 Charité–Universitätsmedizin Berlin, corporate member of Freie Universität Berlin, Humboldt-Universität zu Berlin, and Berlin Institute of Health, Department of Ophthalmology, Berlin, Germany; 4 Charité–Universitätsmedizin Berlin, corporate member of Freie Universität Berlin, Humboldt-Universität zu Berlin, and Berlin Institute of Health, Institute of General Practice, Berlin, Germany; 5 University of Edinburgh, School of Social and Political Sciences, Edinburgh, United Kingdom; 6 Hematology and Hemotherapy Foundation from Amazonas State (HEMOAM), Manaus, Amazonas, Brazil; 7 Labor 28 GmbH, Berlin, Germany; Universidade do Estado do Rio de Janeiro, BRAZIL

## Abstract

**Background:**

Hookworm-related cutaneous larva migrans (HrCLM) is a neglected parasitic skin disease, widespread in resource-poor communities in tropical and subtropical countries. Incidence and risk factors have never been investigated in a cohort study.

**Methodology/Principal findings:**

To understand the seasonal epidemiology of HrCLM, an open cohort of 476 children in a resource-poor community in Manaus, Brazil was examined for HrCLM monthly over a period of 6 months. Monthly prevalence and intensity of infection were correlated with the amount of monthly precipitation. Multivariable Cox regression analysis indicated male sex (hazard ratio [HR] 3.29; 95% confidence interval [CI] 1.95–5.56), walking barefoot on sandy ground (HR 2.30; 95% CI 1.03–5.16), poverty (HR 2.13; 95% CI 1.09–4.17) and age between 10 and 14 years (HR 1.87; 95% CI 1.01–3.46) as predictors of HrCLM. Monthly incidence rates ranged between 0.21 and 1.05 cases per person-year with an overall incidence of 0.52 per person-year.

**Conclusions/Significance:**

HrCLM is a frequent parasitic skin disease in this resource-poor community. Every second child theoretically becomes infected during one year. Boys, 10 to 14 years old, belonging to the poorest households of the community, are the most vulnerable population group. Even in the tropical monsoonal climate of Amazonia there is a considerable seasonal variation with monthly incidence and number of lesions peaking in the rainy season.

## Author summary

Hookworm-worm related cutaneous larva migrans (HrCLM) is a parasitic skin disease caused by hookworm larvae of cats and dogs occurring in many countries with a tropical or subtropical climate. Humans are a biological impasse for these helminths as the larvae cannot pass the basal membrane of the epidermis and hence migrate haphazardly in the skin causing local inflammation and intense itching. In scientific literature HrCLM is generally described as a disease of travellers returning from endemic areas. In contrast, epidemiological data is scanty. In a previous study, we had examined an entire resource-poor neighbourhood in Manaus (Brazil) and showed, that HrCLM is an important individual and public health problem, affecting up to 8% of the population, in particular children. In this study, we followed a cohort of children for six months. We found a significant seasonal variation in incidence and morbidity between dry and rainy season. Extrapolated, every second child in this population will be affected at least once within one year. The longitudinal study design enabled us to validate previously identified risk factors. Children aged 10–14 years, in particular boys, and those walking barefoot on sandy ground had the highest infection rates. Children from the poorest families in the resource-poor community were most vulnerable to HrCLM.

## Introduction

Hookworm-related cutaneous larva migrans (HrCLM) is a neglected tropical skin disease caused by hookworm larvae of cats and dogs such as *Ancylostoma braziliense*, *Ancylostoma caninum* and *Uncinaria stenocephal*a [1]. In humans, these larvae are unable to cross the basal membrane of the epidermis and hence cannot continue their normal development to adult worms. By consequence they haphazardly migrate in the epidermis, producing an elevated linear or serpiginous track. The intense itching leads to important pruritus-associated morbidity such as excoriations and bacterial superinfection of the lesions [2–5].

HrCLM is endemic in many tropical and subtropical countries worldwide [6–9]. Prevalence reached up to 8% in population-based studies with significant variation between sexes and age-groups [2]. Children are affected in particular [2,3]. In semi-arid climates such as in North-eastern Brazil, there is significant seasonal variation in prevalence from 0.2% in the middle of the dry season to 3.1% in the rainy season [4].

Known risk factors are male sex, young age, barefoot walking, poverty and presence of animal faeces on the compound [2,4,10]. In order to determine hazard ratios for previously identified risk factors and verify whether there is seasonal variation of incidence and morbidity in the tropical monsoonal climate of Amazonia, we conducted a longitudinal study with a cohort of 476 children living in a resource-poor community in the outskirts of Manaus, the largest city in Amazonia.

## Methods

### Study area and population

The study was conducted in the resource-poor neighbourhood Nova Vitoria in Manaus, capital of Amazonas State, Brazil. Manaus is situated at 03°06' south latitude and has a tropical monsoonal climate following the Köppen-Geiger classification with a dry season (less than 60 mm precipitation/month) usually in August and heavy monsoon rains during the rest of the year [11]. As many other resource-poor communities in Brazil, the study area was built up without permission of public authorities. This explains the lack of public infrastructure such as health facilities, childcare, paved streets or a sewage disposal system. In consequence many stray dogs and cats roamed through the streets and children were playing unattended on the sandy ground. The population was poor, one third of the households experienced food shortage in the past 12 months. Most of the families had several children. The study area and population have been described in detail previously [2].

### Study design

At baseline all households in the study area were visited. Based on stringent inclusion criteria 92% of the households were admitted to the cross-sectional study. Methods of the cross-sectional study have been published previously [2]. In brief, all household members were examined clinically for HrCLM and environmental, socio-economic and behaviour-related risk factors were documented using pre-tested, structured questionnaires.

**Fig 1:**
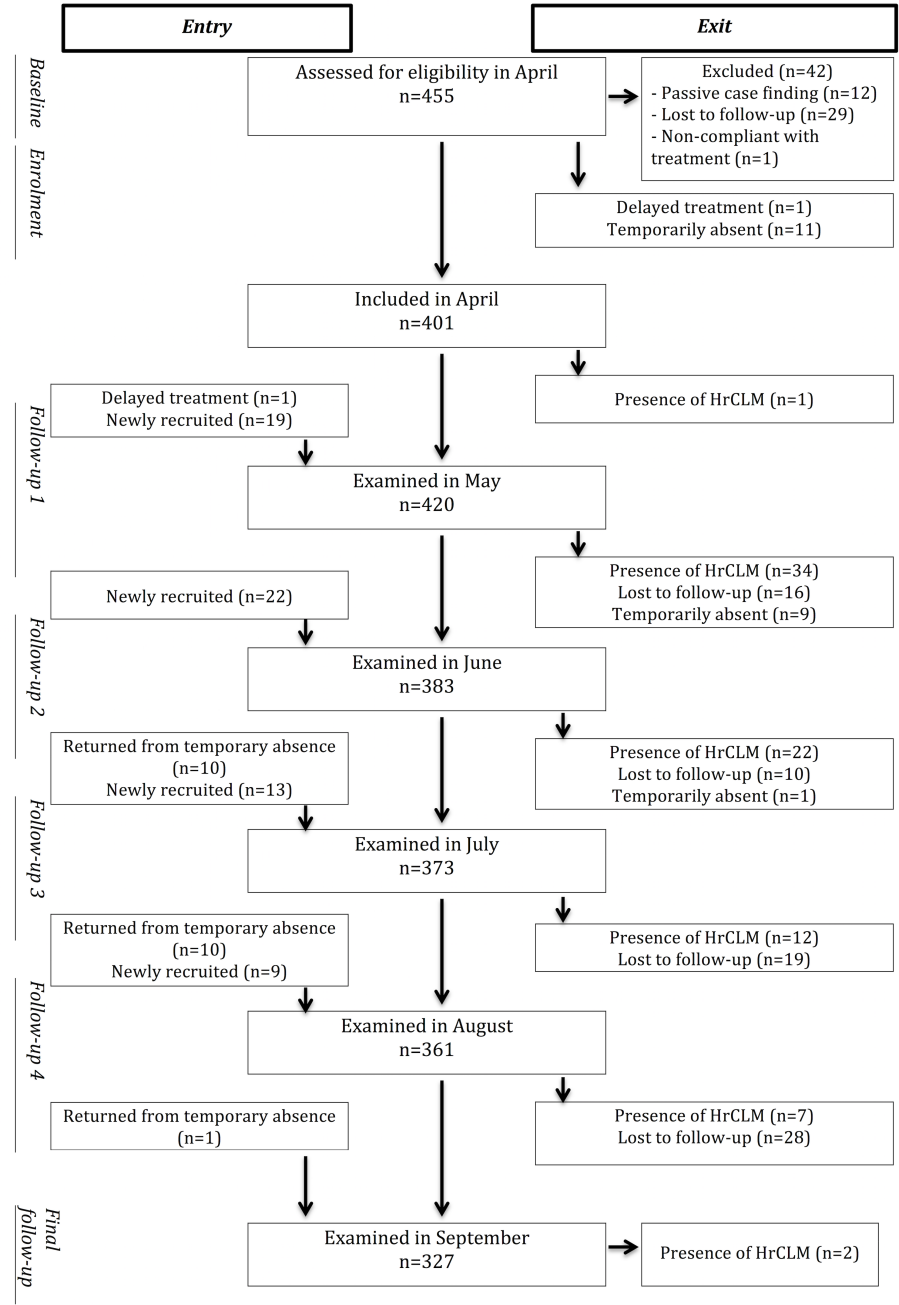
Flowchart of participants in the cohort.

All children of the 262 households were assessed for eligibility for the cohort and then monitored monthly for the presence of new HrCLM (Fig 1). The examination took place in a room where privacy was guaranteed. The whole body surface was examined, only the genital area was spared in case itching was absent. HrCLM was diagnosed clinically when the characteristic slow-progressing, elevated linear or serpiginous track was present [12–14].

Inclusion criteria for the cohort were: residence in the study area, absence of HrCLM-infection at baseline, age under 18 and provision of an informed written consent. Children who were found infected at baseline were included on the day they received treatment with ivermectin or topical thiabendazole. In all but one child, HrCLM lesions had resolved by the next follow-up.

Due to an expected important drop out rate caused by out- and in-migration of whole families and temporary residence of family members in the countryside outside Manaus, we chose an open cohort design. Siblings of participants and children of newly encountered families replaced children who were lost for follow-up in order to keep the number of cohort members stable (Fig 1). Participants who entered the cohort after baseline were examined and interrogated in an identical manner. In case of temporary absence, only the time of presence in the study area between two consecutive follow-ups was analysed.

Participants lost to follow-up before the second examination (n = 29) were excluded from the analysis. To avoid selection bias 12 individuals, who were not identified by active case finding but presented to the team for procurement of treatment, were excluded. One patient was excluded because he refused to be treated with ivermectin (Fig 1).

In order to gather information on reinfections, individuals censored after HrCLM diagnosis were observed further after treatment (S1 Fig). These data were included in the analysis of monthly incidence and prevalence, and for analysis of clinical characteristics.

Episodes of HrCLM at recruitment were documented and included in the analysis of clinical characteristics and monthly prevalence, if the prerequisite of residency for more than 2 months in the study area was fulfilled.

For the calculation of monthly incidence rates only participants without HrCLM or participants that had been treated in the preceding month were included.

The study started in April 2009 (rainy season) and ended in September 2009 (which in 2009 was still part of the dry season).

### Ethical considerations

The study was approved by the Ethical Committee of the Fundação de Medicina Tropical–Amazonas (FMT-AM). Informed written consent was obtained from each participant’s legal guardian. Every individual with HrCLM was offered free treatment independently whether he or she participated in the study. Treatment consisted of ivermectin (Ivermec, Uci-farma, São Paulo, Brazil) given as single oral dose (200 μg/kg). In the case of children <5 years or <15 kg, thiabendazole 5% (Tiadol, Bunker Indústria Farmacêutica Ltda., São Paulo, Brazil) was topically applied 3 times a day for one week [2].

### Statistics

Data were entered in Microsoft Office Access 2007, cleaned for entering errors and analysed with PASW Statistics Version 18.0 (SPSS Inc., Chicago, USA). Missing data were assumed to be missing at random and were marked in the analysis. A wealth score was formed out of household assets using principal component analysis as previously described [2,15]. Income and age were categorized similar to previous population-based studies to allow comparison [2,4,10,16].

Time to infection was defined as the time between the date of inclusion in the cohort and the date of diagnosis of HrCLM, date of last examination before lost to follow-up, or date of last examination at the end of the study (right censoring), whichever occurred first. In case of temporary absence, only the time between consecutive follow-ups was included. Lost to follow-ups were compared to those who were censored by event or end of the study using chi-square-test or likelihood ratio where appropriate.

For bivariable risk factor analysis, hazard ratios (HR) were calculated together with 95% confidence intervals (95% CI) using a Cox proportional hazards model.

For multivariable risk factor analysis, all variables that showed weak evidence of an association with HrCLM (p<0.1) were entered into a stepwise Cox regression. Only significant variables (p<0.05) remained in the model. We calculated standard errors and 95% CI to identify multicollinearity and removed variables where necessary. We used log-minus-log function to check if proportional hazard assumption was satisfied.

Kaplan-Meier curves were used to visualize event-free periods and differences in incidence between sexes, age groups, frequency of barefoot walking and wealth strata.

We calculated Population Attributable Fraction (PAF) for independent risk factors practically amenable to intervention. As only one risk factor was amenable to intervention, we used Levins unadjusted equation: {p_e_(RR-1)}/ {p_e_(RR-1)+1} with p_e_ being the proportion of the population, which is exposed to the risk factor [17,18]. RR was calculated as a rate ratio.

Incidence rate and prevalence were determined monthly. Seasonal changes in prevalence, clinical presentation (superinfection, number of lesions, site of infection) and total monthly precipitation in Manaus were correlated using Spearman´s rank correlation with a two-tailed significance level of p<0.05. Climate data were obtained from the International Institute of Meteorology of Brazil (INMET).

The overall incidence rate was extrapolated from the number of patients divided by the accumulated time to HrCLM infection. Correspondingly, reinfection rate was calculated for the subgroup of patients who were infected at the first examination and were included after treatment. Monthly incidence rates were obtained by dividing the number of HrCLM-infections by the number of followed children during the corresponding time period multiplied by 12 assuming an exact 1-month-period between two follow-ups. Corresponding 95% CI were calculated by a Poisson regression.

## Results

During the 6 months of the study, a total of 476 children living in 209 households were included with a median time of follow-up of 149 days (range 13–166), which amounted to 54,938 person-days at risk. The median age at entry was 6 years (range 0–15) and the majority of the children were girls (52%). After a median time of 65.5 days (range 22–132), 68 children (14.3%) were lost to follow-up. Comparing their characteristics, there was no significant difference except for the proportion of barefoot walking on sandy ground (Table 1).

**Table 1:** Comparison between participants lost to follow-up and censored by event or end of study (N = 476).

Characteristic	Total no. (%)	Lost to follow up (%)	Censored by event or end of study (%)	2-sided p-value *
**Demography**				
Male	228 (47.9)	26 (38.2)	202 (49.5)	0.083
Female	248 (52.1)	42 (61.8)	206 (50.5)	
Age ≤ 4 years	174 (36.6)	32 (47.1)	142 (34.8)	0.147
5–9 years	188 (39.5)	22 (32.4)	166 (40.7)	
10–14 years	102 (21.4)	11 (16.2)	91 (22.3)	
15–18 years	12 (2.5)	3 (4.4)	9 (2.2)	
**Socioeconomic characteristics**				
Income < 1 minimum wage †, ‡	133 (28.5)	18 (26.5)	115 (28.9)	0.130
= 1 minimum wage	169 (36.3)	19 (27.9)	150 (37.7)	
> 1 minimum wage	164 (35.2)	31 (45.6)	133 (33.4)	
Wealth score †, §				
Low	197 (43.2)	33 (48.5)	164 (42.3)	0.623
Intermediate	151 (33.1)	20 (29.4)	131 (33.8)	
High	108 (23.7)	15 (22.1)	93 (24.0)	
**Behaviour**				
Walking always/regularly †				
Barefoot outdoors	61 (12.9)	5 (7.5)	56 (13.8)	0.154
With sandals/shoes outdoors/ not walking at all (babies)	431 (87.1)	62 (92.5)	351 (86.2)	
Walking on sandy ground †				
Always barefoot	94 (20.0)	7 (10.6)	87 (21.5)	0.001
Sometimes barefoot	281 (59.8)	35 (53.0)	246 (60.9)	
Never barefoot	95 (20.2)	24 (36.4)	71 (17.6)	
Practicing soccer †	172 (36.5)	19 (27.9)	153 (38.0)	0.208
Other sport	39 (8.3)	8 (11.8)	31 (7.7)	
No sport	260 (55.2)	41 (60.3)	219 (54.3)	
Sport barefoot on sand †	170 (36.2)	18 (26.5)	152 (37.8)	0.105
Never barefoot/not on sand	40 (8.5)	9 (13.2)	31 (7.7)	
No sport	260 (55.3)	41 (60.3)	219 (54.5)	
**Environment**				
Faeces found on compound †	65 (14.7)	5 (7.8)	60 (15.8)	0.094
No faeces found	378 (85.3)	59 (92.2)	319 (84.2)	
**Health**				
Infection at first examination	64 (13.4)	6 (8.8)	58 (14.2)	0.228
No infection	412 (86.6)	62 (91.2)	350 (85.8)	

*For comparison of „Lost to follow-up”and „Censored by event or end of the study”group.

†Missing observations.

‡Minimum wage in 2009: 465 R$ ≈ 220$.

§For definitions see methods.

**Fig 2:**
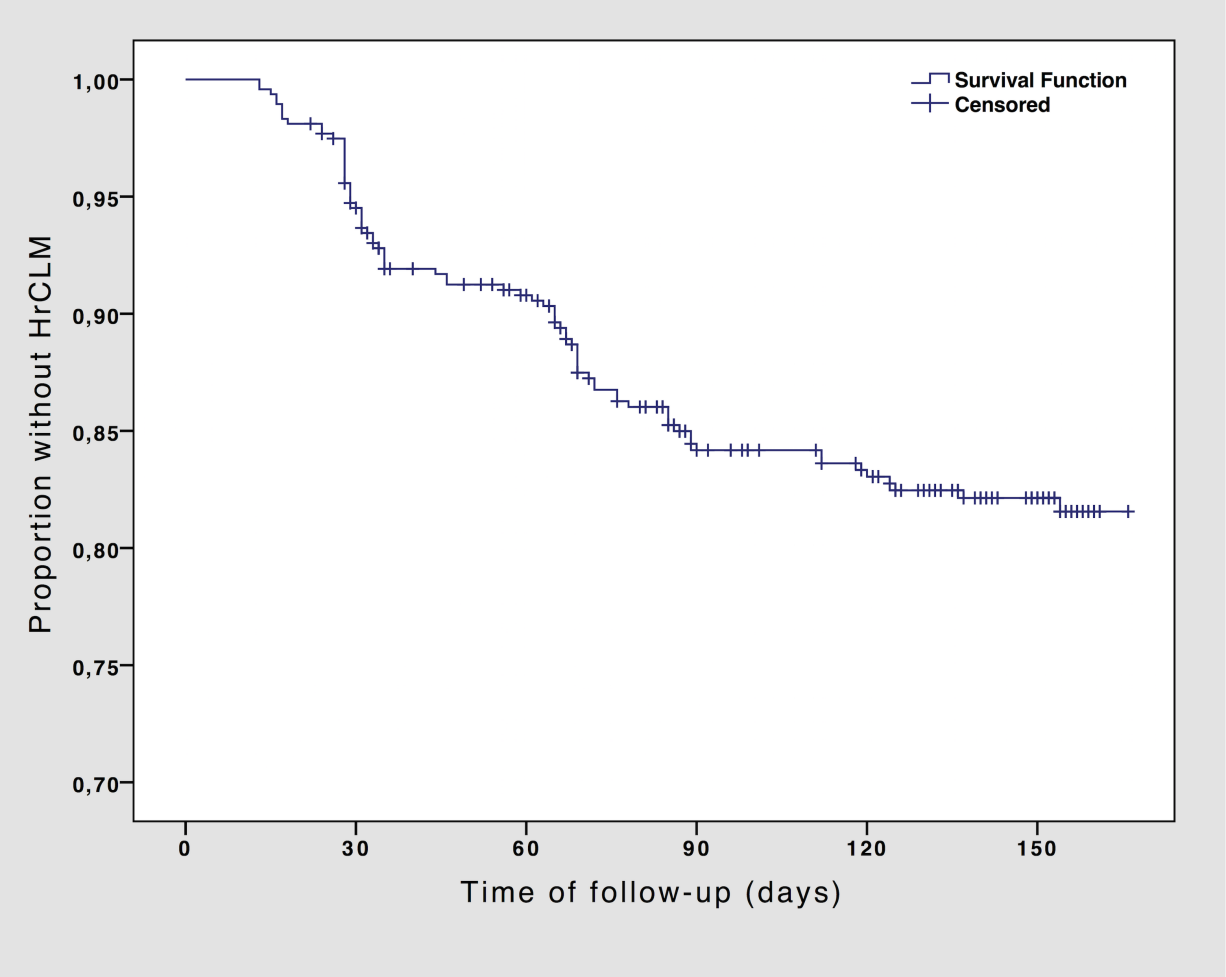
Kaplan-Meier-curve for overall HrCLM-free proportion.

The Kaplan-Meier estimated HrCLM-free proportion after 90 days and after 166 days (at the end of the study) was 84% and 82%, respectively (Fig 2). Mean time to HrCLM infection was 146.4 days (95% CI 142.3–150.5). There was no difference in mean time to HrCLM infection between recruitment at baseline or later (145.6 days, 95% CI 141.1–150.0 days and 139.3 days, 95% CI 128.5–150.1 days, respectively, p = 0.462).

A total of 161 episodes of HrCLM were identified. Only 27 children (5.7%) accounted for 66 (41.0%) of all episodes by having several (up to 4) episodes of HrCLM. During follow-up, 78 children developed HrCLM resulting in an overall incidence rate of 0.52 per person-year.

**Fig 3:**
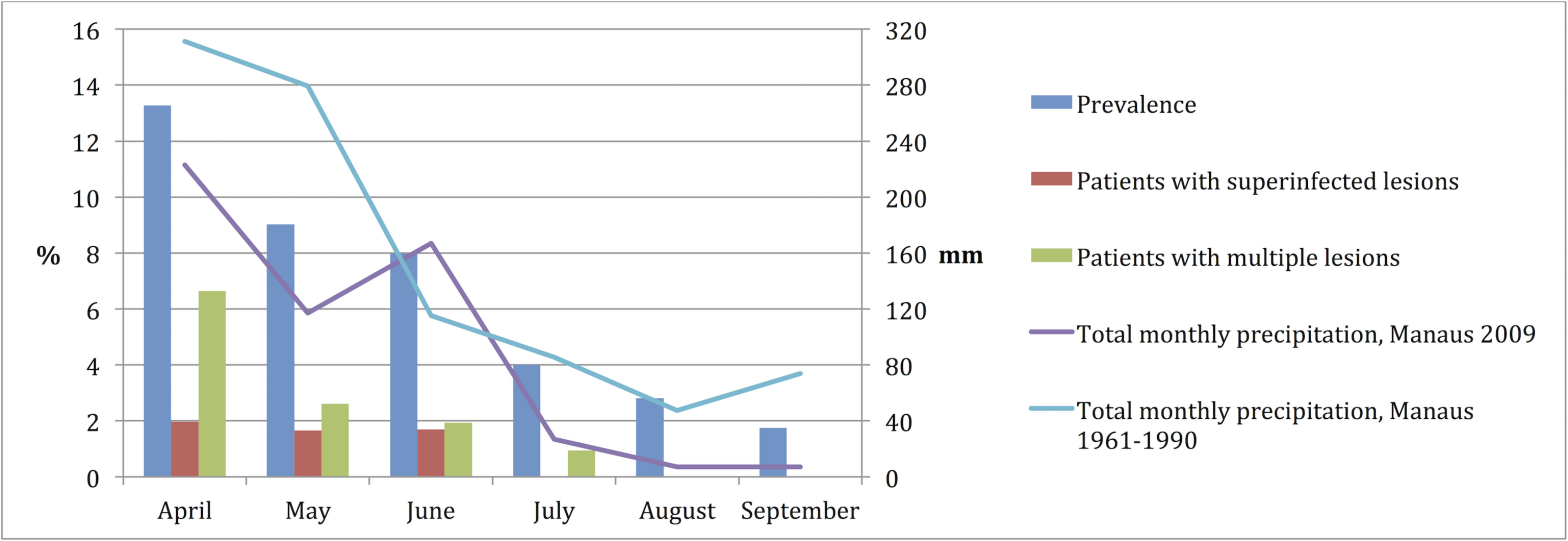
Precipitation, prevalence and clinical characteristics by month.

Reinfection rate of children who were infected before inclusion in the cohort (n = 64) was more than twice as high with 1.08 per person-year. Monthly incidence rates and prevalence decreased every month from the rainy to the dry season (Table 2; Fig 3).

**Table 2:** Incidence of hookworm-related cutaneous larva migrans (HrCLM).

	Person-years at risk	Patients with HrCLM	Incidence per person-year	(95% CI)
**By month**				
April-May	33.42 *	35	1.05	0.76–1.48
May-June	32.67 *	27	0.83	0.58–1.22
June-July	34.25 *	16	0.47	0.30–0.78
July-August	34.83 *	12	0.34	0.21–0.63
August-September	33.33 *	7	0.21	0.11–0.46
**Overall**	150.5	78	0.52	0.42–0.65
**Reinfection Rate** †	17.53	19	1.08	0.71–1.73

* Approximated data, assuming an exact 1-month-period between two follow-ups.

† Calculated with patients who were infected at baseline and were included after treatment; see study design.

Clinical characteristics showed seasonal differences. During the rainy season in April, every second affected child had multiple lesions and lesions were found all over the body. During the dry season in contrast, only single lesions were encountered and lesions predominantly occurred on hands and feet. Superinfected lesions were only found from April to June (Table 3 and Fig 3).

**Table 3:** Clinical characteristics of study participants at different times of examination.

	April (N = 407)		May (N = 421)		June (N = 414)		July (N = 424)		August (N = 427)		September (N = 400)	
Characteristic	n	%	n	%	n	%	n	%	n	%	n	%
Children with HrCLM*	54	13.3	38	9.0	33	8.0	17	4.0	12	2.8	7	1.8
Children with superinfected lesions †	8	14.8 ‡	7	18.4 ‡	7	21.2 ‡	0	0	0	0	0	0
Number of lesions per child												
1	27	50.0 ‡	27	71.1 ‡	25	75.8 ‡	13	76.5 ‡	12	100 ‡	7	100 ‡
2	18	33.3 ‡	3	7.9 ‡	3	9.1‡	3	17.7 ‡	0	0	0	0
≥3	9	16.7 ‡	8	21.1 ‡	5	15.2 ‡	1	5.9 ‡	0	0	0	0
Affected body areas §,#			*¶*						*¶*			
Hand	5	7.7	2	4.7	3	8.3	1	5.6	1	9.1	1	14.3
Arm	6	9.2	1	2.3	1	2.8	1	5.6	0	0	0	0
Trunk	5	7.7	6	14.0	1	2.8	1	5.6	0	0	0	0
Buttock	6	9.2	4	9.3	3	8.3	1	5.6	0	0	0	0
Head	1	1.5	1	2.3	0	0	0	0	0	0	0	0
Leg	5	7.7	2	4.7	3	8.3	0	0	1	9.1	0	0
Foot	35	53.9	23	53.5	21	58.3	12	66.7	8	72.7	5	71.4
Toe	2	3.1	4	9.3	4	11.1	2	11.1	1	9,1	1	14.3

*Hookworm-related cutaneous larva migrans.

†Pustules or suppuration.

‡Percent of children with HrCLM.

§Multiple topographic affection possible.

¶Missing observations.

#Percent of affected body areas.

The decreases in prevalence and infection intensity (multiple infections per person) were correlated with the decreasing amount of monthly precipitation (rho = 0.928, p = 0.008, and rho = 0.941, p<0.001 respectively).

Bivariable risk factor analysis revealed 10–14 years of age, a low wealth score, practicing soccer (soccer is usually played barefoot), walking barefoot on sandy ground and HrCLM-infection at recruitment as predictors for HrCLM-infection during the follow-up period. The most potent predictor was male sex (Table 4).

**Table 4:** Predictors of hookworm-related cutaneous larva migrans in a cohort of 476 children.

Characteristic	Person-years at risk	HrCLM*	Incidence per person-year	Crude Hazard Ratio (95% CI)	2-sided p-value	Adjusted Hazard Ratio (95% CI)	2-sided p-value
**Demography**							
Male	65.90	57	0.86	3.41 (2.04–5.69)	<0.001	3.29 (1.95–5.56)	<0.001
Female	84.62	21	0.25	1 †		1 (reference)	
Age ≤ 4 years	55.87	21	0.38	1 †		1 (reference)	
5–9 years	60.48	28	0.46	1.28 (0.72–2.27)	0.40	1.07 (0.59–1.94)	0.824
10–14 years	30.24	28	0.93	2.28 (1.27–4.08)	0.006	1.87 (1.01–3.46)	0.045
15–18 years	3.92	1	0.26	0.78 (0.11–5.83)	0.811	0.65 (0.09–4.91)	0.674
**Socioeconomic characteristics**							
Income < 1 minimum wage ‡, §	43.46	22	0.51	1.27 (0.70–2.32)	0.429		
= 1 minimum wage	51.85	34	0.66	1.62 (0.94–2.78)	0.084		
> 1 minimum wage	51.95	21	0.40	1 †			
Wealth score ‡, *¶*							
Low	60.51	41	0.68	2.15 (1.11–4.18)	0.024	2.13 (1.09–4.17)	0.027
Intermediate	48.81	23	0.47	1.51 (0.74–3.10)	0.259	1.47 (0.71–3.04)	0.294
High	35.79	11	0.31	1 †		1 (reference)	
**Behaviour**							
Walking always/regularly ‡							
Barefoot outdoors	17.89	14	0.78	1.57 (0.88–2.80)	0.126		
With sandals/shoes outdoors/ not walking at all (babies)	132.09	64	0.48	1 †			
Walking on sandy ground ‡							
Always barefoot	27.90	25	0.90	3.24 (1.51–6.94)	0.002	2.30 (1.03–5.16)	0.043
Sometimes barefoot	89.48	44	0.49	1.66 (0.81–3.42)	0.168	1.70 (0.81–3.56)	0.158
Never barefoot	31.22	9	0.29	1 †		1 (reference)	
Practicing soccer ‡	51.39	38	0.74	1.86 (1.17–2.95)	0.009		
Other sport	11.30	5	0.44	1.08 (0.42–2.74)	0.88		
No sport	86.14	34	0.39	1 †			
Sport barefoot on sand ‡	50.39	38	0.75	1.89 (1.19–3.00)	0.007		
Never barefoot/not on sand	11.88	5	0.42	1.04 (0.41–2.65)	0.939		
No sport	86.14	34	0.39	1 †			
**Environment**							
Faeces present on compound ‡	21.02	11	0.52	1.01 (0.53–1.92)	0.972		
No faeces present	123.45	63	0.51	1 †			
**Health**							
Infection at first examination	17.53	19	1.08	2.34 (1.40–3.93)	0.001		
No infection	132.99	59	0.44	1 †			

*Number of children with Hookworm-related cutaneous larva migrans.

† Reference category.

‡ Missing observations.

§ Minimum wage in 2009: 465 R$ ≈ 220$.

¶ For definitions see methods.

**Fig 4:**
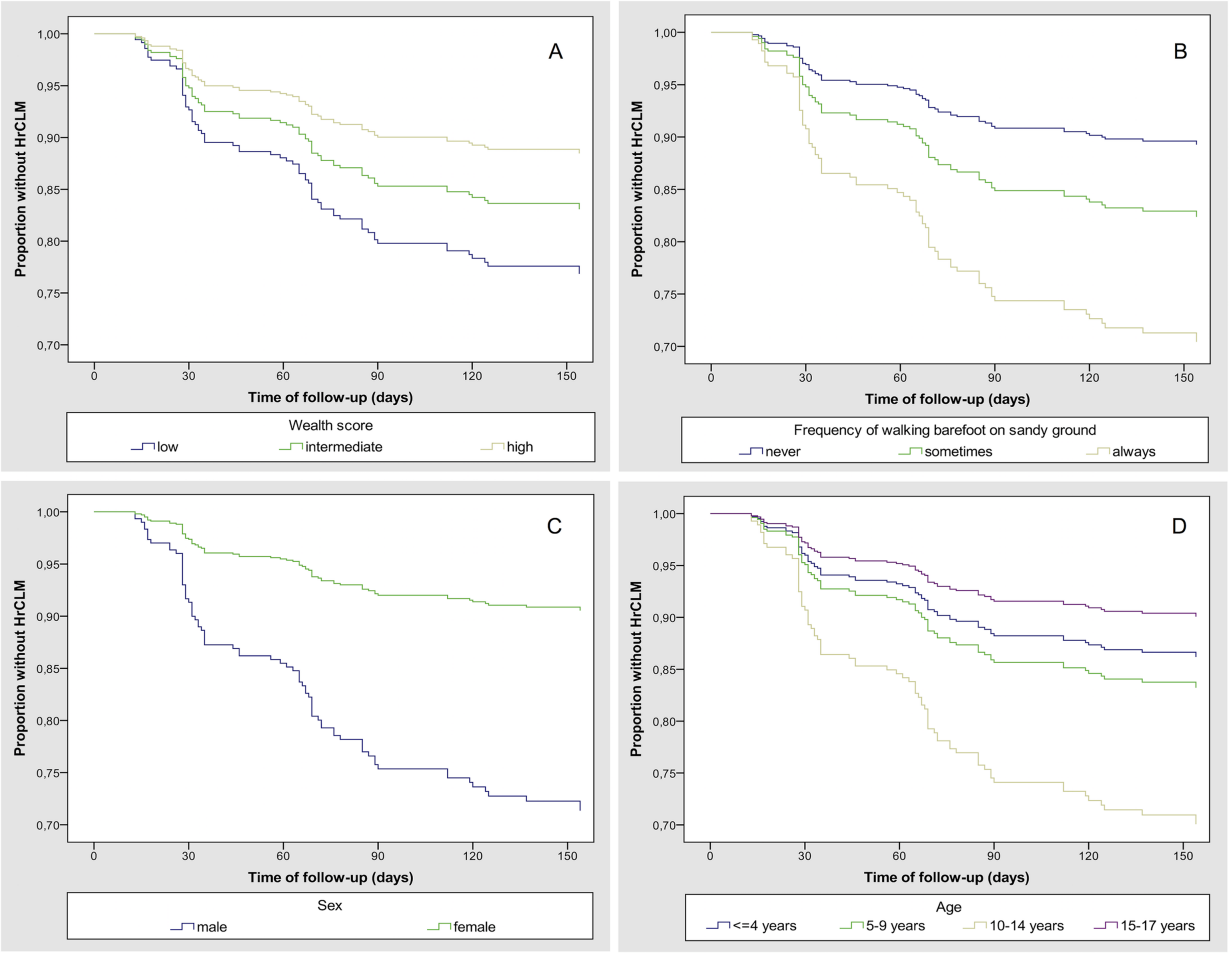
Stratified Kaplan-Meier curves for HrCLM-free proportion. (A) wealth (B) barefoot walking (C) sex (D) age.

In the adjusted multivariable model age, sex, low wealth and barefoot walking remained as independent risk factors visualized in Kaplan-Meier curves (Fig 4). The PAF of walking barefoot on sandy ground was 45%.

## Discussion

In this longitudinal study on the occurrence and determinants of HrCLM, we could show that higher infection rates correlate with larger amounts of precipitation even in a tropical monsoonal climate where soil remains wet the whole year round.

Development and transmission of nematode larvae depend on a favourable climate. High soil moisture and atmospheric humidity enhance development and survival of eggs and larvae [19]. Rain furthermore disperses larvae and eggs over a larger soil surface [20]. Consequently, climate defines the endemic areas and may cause seasonal variation. Seasonal variation in prevalence with positive correlation to precipitation has been shown for the semi-arid climate of Northeast Brazil [4].

Additionally, variations in climate may have an indirect influence on prevalence through a change in behaviour of the population e.g. regarding sports or use of protective footwear.

For the first time, our study showed that precipitation correlated with disease intensity. This is not surprising as more infective stage larvae do not only increase infection risk on the population but also on the individual level.

Interestingly, seasonal variation extended to the topographic localisation of infection. While untypical localisations such as the trunk, buttock or head were found in up to one fourth of infections during the rainy season, only feet and hands were affected at the last examination during dry season. These body areas are often uncovered, have most direct contact with the soil and therefore get infected the whole year round. Body areas with less exposure to the soil seem to get infected regularly only in case of a higher larval burden in the soil. Similar results were seen in semi-arid Northeast Brazil where untypical localisations accounted for more than half of the lesions during rainy season [4,16].

### Incidence

So far, an incidence rate of HrCLM has never been determined in an appropriately designed study. Calculated monthly it showed a seasonal variation between a maximum of 1.05 per person-year during rainy season and a minimum of 0.21 per person-year during dry season.

The overall incidence rate was 0.52 cases per person-year. That means that on the average every second child will get infected with HrCLM within one year, if the observed six months are representative for the whole year.

The reinfection rate was even higher (1.08 per person-year). HrCLM-infections are not distributed homogenously throughout the child population; but obviously, children with a certain high-risk profile get infected several times a year.

Considering the possible implications of such an infection including pruritus, sleep disturbance, impaired school performance and superinfection, this is a massive problem within the child population[5,21].

### Risk factors

Most of the known risk factors identified in our previous cross-sectional study in Manaus and other studies in Northeast Brazil, could be confirmed by the present cohort study [2,4,10]. Male sex was the strongest predictor for HrCLM-infection with a more than three times higher risk in boys than in girls. Children between 10 and 14 years had a nearly two-fold higher risk to get infected than children of the youngest age group. Both might be due to gender-related behaviour or the extent of parental surveillance, as hypothesised earlier [4]. Generally, boys may spend more time outdoors, have more contact with the soil when playing and are less attended by their parents. These characteristics may be more present when children grow older, which might explain in part the age-related differences in hazard ratios.

Walking barefoot especially on sandy ground may also be a risk factor in travellers [6]. In endemic areas, the frequency of using protective footwear clearly influences the risk of infection [2]. The estimated PAF related to barefoot walking on sandy ground was 45%, meaning that nearly half of the infections with HrCLM could have been prevented if all children wore shoes [17]. Even plastic sandals, which are the typical footwear in this area, can prevent infection [2].

HrCLM is a poverty-related disease. Poverty-related living conditions with stray dogs and cats, unpaved streets and many unattended children playing in the streets create a beneficial environment for the transmission of hookworm larvae. More than half of the study households had only one minimum wage or less to their disposition [2]. But even within this poor community, poverty was an independent predictor for the acquisition of HrCLM. This corroborates the results of our cross-sectional study as well as observations from other neglected tropical diseases [2,22,23].

The presence of animal faeces on the compound was the only independent risk factor identified in the previous cross-sectional study that could not be confirmed by this study. This might be due to the different composition of the study population with only children, who might spent more time outside the compound while playing with other children.

### Policy recommendations

Accordingly, the only deducible measure for disease control in the study area is prevention of barefoot walking. However, just providing shoes might not be sufficient similar to what we previously observed in a study on tungiasis control [24]. The underlying causes are much more complex. Even good parental knowledge about disease etiology couldn´t prevent HrCLM-infection [2,21]. Mothers feel unable to look out for their children [21]. Incidence was highest in boys aged 10–14 who probably are the less attended children in a poor household. Furthermore, the disease burden was highest among the poorest of the poor. In this way, HrCLM is a neglected disease acquired by the most neglected parts of the population. Only by changing social circumstances and health education of parents and children, preventive behaviour may be established.

### Limitations

There may have been a selection bias in favour of younger children because of school attendance and work during visiting hours in the daytime. For security reasons visiting hours could not be extended beyond 6 p.m. Due to an exhaustive sampling strategy and high participation rate, we did obtain, however, a representative sample of the paediatric daytime population, which most likely is predominantly exposed in the study area.

Although children carry the biggest part of disease burden, the results cannot be translated to the whole population as no adults participated. [2,4,10].

We observed a differential loss of participants (Table 1). This might have led to an overestimation of events in the group exposed to “walking barefoot on sandy ground”. Otherwise we have no reasons to believe that missing data biased the results.

The absence of treatment in the study area may have led to more HrCLM-infections in April and biased therefore the changes in disease intensity (multiple lesions per person) and severity (superinfection). However, the decrease in disease intensity continued over the whole study period and the correlation with monthly precipitation was significant.

Incidence rate was calculated with person-time and referred to a whole year even though the probability of disease is not constant during the study period. The observation period, however, included three months of the dry and three months of the raining season and may therefore be representative for the whole year.

### Conclusion

In conclusion, the prevalence of HrCLM showed seasonal variation and was correlated with precipitation and disease intensity. Overall incidence rate among children was as high as 0.52 per person-year. Independent risk factors were male sex, age, walking barefoot on sandy ground and extreme poverty.

## Supporting information

S1 Fig Flowchart of participants in the cohort extended by treated and further observed cases. (TIFF)

S1 Database Study database. (SAV)
