# Supplementary figures and images for: Epidemiology and morbidity of hookworm-related cutaneous larva migrans (HrCLM): Results of a cohort study over a period of six months in a resource-poor community in Manaus, Brazil
Source: PLoS Negl Trop Dis. 2018 Jul 19;12(7):e0006662. doi: 10.1371/journal.pntd.0006662 (PMC6067763; doi:10.1371/journal.pntd.0006662)

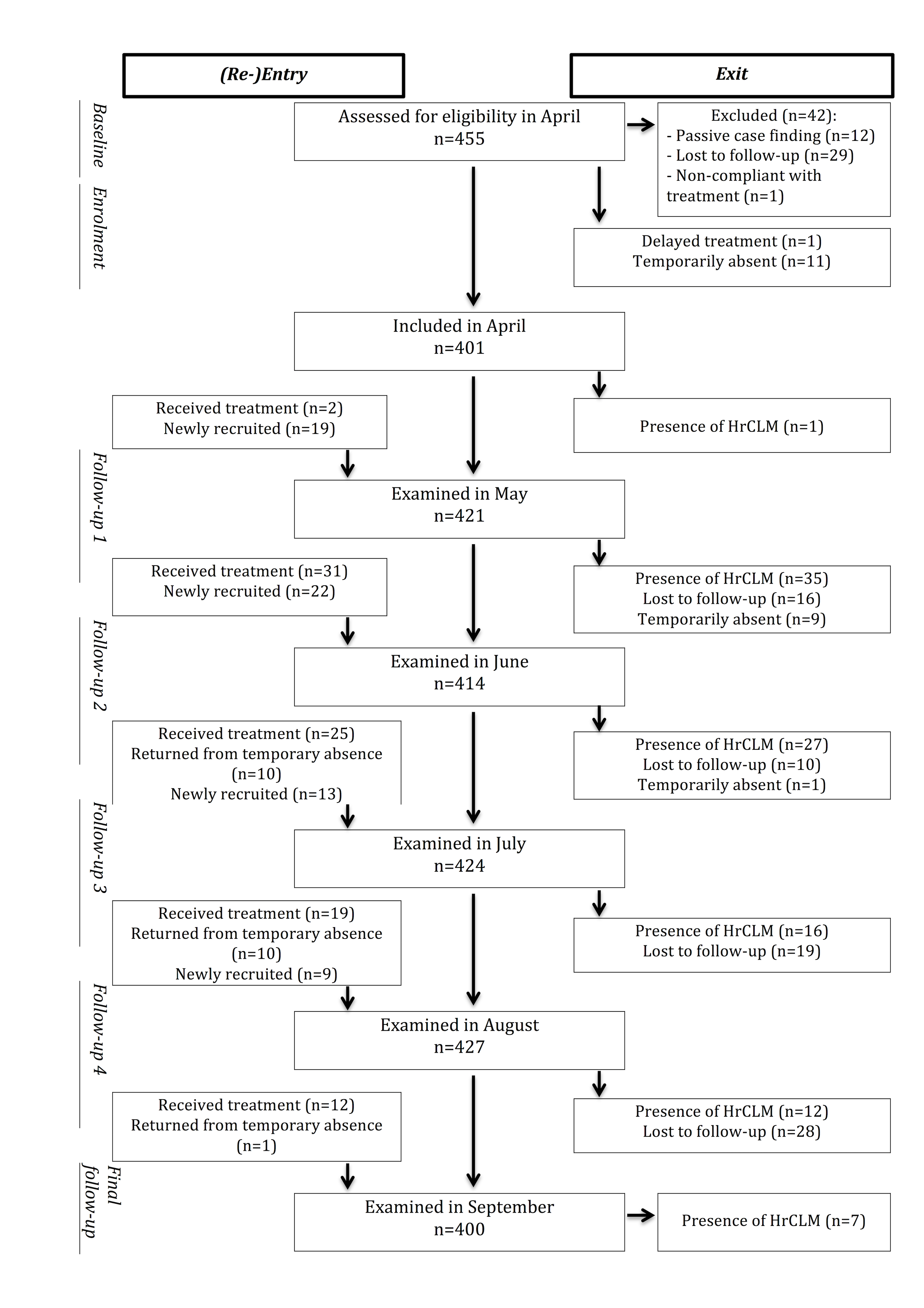

Supplement: S1 Fig — (TIFF) [file pntd.0006662.s001.tiff]
